# Decoding endophytic microbiome dynamics: engineering antagonistic synthetic consortia for targeted fusarium suppression in monoculture regimes

**DOI:** 10.1093/hr/uhaf286

**Published:** 2025-10-16

**Authors:** Hongling Qin, Leyan Zhang, Zhongxiu Rao, Xiaomeng Wei, András Táncsics, Rong Sheng, Yi Liu, Anlei Chen, Cheng Fang, Fengqiu Huang, Pan Long, Baoli Zhu

**Affiliations:** Institute of Subtropical Agriculture, Chinese Academy of Sciences, Changsha 410125, China; College of Agronomy, Hunan Agricultural University, Changsha 410128, China; Hunan Soil and Fertilizer Institute, Changsha 410125, China; College of Resources and Environment, Northwest Agriculture and Forestry University, Yangling 712100, China; Department of Molecular Ecology, Hungarian University of Agriculture and Life Sciences, H-2100 Gödöllő, Hungary; Institute of Subtropical Agriculture, Chinese Academy of Sciences, Changsha 410125, China; Institute of Subtropical Agriculture, Chinese Academy of Sciences, Changsha 410125, China; Institute of Subtropical Agriculture, Chinese Academy of Sciences, Changsha 410125, China; Institute of Subtropical Agriculture, Chinese Academy of Sciences, Changsha 410125, China; Hunan Soil and Fertilizer Institute, Changsha 410125, China; College of Agronomy, Hunan Agricultural University, Changsha 410128, China; Institute of Subtropical Agriculture, Chinese Academy of Sciences, Changsha 410125, China

## Abstract

Biological control leveraging endophytic microbes represents a promising eco-friendly strategy to mitigate soil-borne diseases, yet the efficacy and mechanistic underpinnings of synthetic microbial communities (SynComs) derived from plant endophytes remain poorly understood. This study employed a holistic approach—integrating field sampling, microbial profiling, and functional validation—to investigate the dynamics of edible lily (*Lilium*) microbiomes under continuous cropping and develop targeted SynComs against *Fusarium oxysporum*. Metacommunity analysis revealed that prolonged monoculture co-enriched both potentially beneficial taxa (e.g. *Pseudomonas*, *Bacillus*) and pathogenic *Fusarium*, reflecting a dynamic equilibrium where naturally recruited antagonists were insufficient to prevent pathogen dominance, while increasing the complexity of endophytic co-occurrence networks. Keystone bacterial lineages, including *Burkholderiaceae* and *Pseudomonas*, emerged as critical stabilizers of the endosphere microbiome. Notably, 50% of endogenous bacterial taxa exhibited rhizospheric origins, contrasting with fungal communities where <10% derived from soil—a finding underscoring host-specific filtering mechanisms. Through systematic isolation and combinatorial testing, we engineered SynComs combining core antagonistic strains (*Rhizobium*, *Methylobacterium*, *Talaromyces*) with auxiliary microbes. Fungal-integrated SynComs outperformed bacteria-only consortia in plant growth promotion and pathogen suppression. By bridging fundamental microbial ecology with translational agriculture, our findings establish SynComs as scalable tools for sustainable soil health management, reducing reliance on synthetic fungicides while addressing the yield-limiting challenges in continuous cropping systems.

## Introduction

Continuous cropping (CF), defined as the prolonged cultivation of the same crop or related species on identical agricultural plots, frequently results in soil degradation, heightened disease vulnerability, and yield reduction [1, 2]. These detrimental effects persist even when employing appropriate agronomic management techniques [3]. Notably, approximately 70% of taproot species encounter substantial cultivation challenges associated with this practice [4]. Elucidating the underlying mechanisms of these obstacles and developing effective mitigation strategies has consequently emerged as a critical focus in global agricultural research.

The proliferation of soil-borne pathogens constitutes a principal contributory factor, arising from microbial community shifts characterized by pathogenic proliferation, beneficial microbe depletion, and diminished microbial diversity [5]. These alterations collectively impair soil’s natural pathogen suppression capacity [6]. Nevertheless, the fundamental mechanisms through which continuous cropping exerts adverse effects on taproot species remain inadequately characterized.

Plant species employ sophisticated signalling mechanisms to detect pathogenic threats and selectively recruit beneficial soil microbiota. These symbiotic relationships facilitate pathogen control, nutrient acquisition enhancement, and growth promotion [7, 8]. Comprehensive understanding and strategic manipulation of plant microbiomes are therefore paramount for developing next-generation phytopathogen management approaches to enhance crop health and productivity. Microbiome assembly is influenced by multiple biotic and abiotic factors [9, 10], with pathogenic invasion representing a particularly significant biotic stressor [11, 12]. Previous investigations have demonstrated that roots of pathogen-infected plants exhibit chemotactic recruitment of beneficial microbes, potentially facilitating plant rescue responses or transgenerational protection [13, 14]. Beneficial microbiota contribute to disease suppression through multiple mechanisms including immune system priming, antibiotic production, and ecological niche competition [15]. While existing research has predominantly focused on rhizospheric and phyllospheric microbiomes, systematic understanding of microbiome structural and functional relationships across rhizospheric, phyllospheric, and endospheric compartments, particularly under pathogen pressure induced by continuous cropping, remains incomplete.

Biological control methods are widely regarded as environmentally sustainable due to their pathogen-specific targeting, which preserves beneficial organisms and maintains ecological equilibrium while minimizing resistance development [16, 17]. However, field efficacy of many biocontrol agents remains suboptimal. Endophytic microorganisms present particularly promising biocontrol candidates owing to their intimate symbiotic relationships with host plants [18]. Through evolutionary co-adaptation, plants and endophytes have developed mutualistic associations where endophytes benefit from host colonization while conferring disease resistance advantages, notwithstanding initial plant defence responses [19, 20]. Practical implementation of endophyte-based biocontrol strategies remains challenging due to inconsistent efficacy, with single-strain applications often failing due to competitive exclusion by indigenous microbiota [21]. Synthetic microbial communities (SynComs) offer potential advantages over monoculture inoculants through enhanced ecological resilience in non-sterile environments [22]. Nevertheless, the protective efficacy of SynComs derived from natural microbiomes against pathogens remains unverified, and standardized methodologies for constructing functional SynComs require further development.

This investigation examines microbial community dynamics across bulk soil, rhizosphere, root episphere, bulb episphere, and endosphere compartments during continuous lily cultivation. Through isolation of indigenous strains, we develop a SynCom formulation for pathogen suppression. We hypothesize that: (i) plants selectively recruit antagonistic endophytes as defence mechanisms against pathogenic infection, and (ii) SynComs exhibit superior efficacy compared to single-strain inoculants.

## Results

### Diversity of rhizosphere microbiome and endophytes in lily bulbs

**Figure 1:**
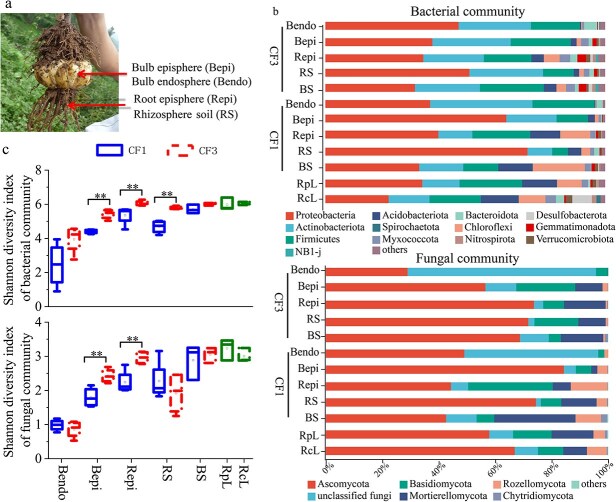
Diversity of bacterial and fungal communities in Edible Lily. (a) Diagram illustrating the various aboveground compartments of an edible lily, including Bulk Soil (BS), Rhizosphere Soil (RS), Root Episphere (Repi), Bulb Episphere (Bepi), and Endosphere (Bendo). Four biological replicates per treatment (*n* = 4). (b) Histogram showing the relative abundance of bacterial and fungal communities at the phylum level. (c) Shannon diversity indices of bacterial and fungal communities across the five compartments after one year (CF1, solid line) and three years (CF3, dashed line) of continuous edible lily cropping. RcL and RpL, bulk soils planted with rice and rapeseed after 3 years of continuous edible lily cropping, respectively. Vertical bars denote the standard deviation from the mean (*n* = 4).

High-throughput sequencing yielded 4 794 490 bacterial (16S rRNA) and 4 887 350 fungal (ITS) high-quality reads, clustered into 5641 bacterial and 4872 fungal OTUs (97% similarity threshold). Dominant phyla included *Proteobacteria*, *Actinobacteriota*, and *Firmicutes* (bacteria) and *Ascomycota*, *Basidiomycota* (fungi) across all compartments (Fig. 1b). Lily continuous cropping significantly increased dissolved organic carbon (DOC), available phosphorus (AP), and available potassium (AK) content. These nutrient alterations substantially restructured soil microbial communities under continuous cropping (CF) conditions (Table S1; Fig. S1). Alpha diversity (Shannon index) demonstrated compartment-specific responses: CF increased bacterial diversity in rhizosphere soil (RS), root episphere (Repi), and bulb episphere (Bepi), while fungal diversity only increased in Repi and Bepi (Fig. 1c, Table 1). However, the alpha diversity indices in the rotation treatments (RcL, rice after lily; RpL, rapeseed after lily) showed no significant differences compared to either the first-year lily field (CF1) or the third-year continuous lily field (CF3) (Fig. 1c).

**Table 1:** Two-way analysis of variance (ANOVA) was used to analyse the alpha diversity of bacterial and fungal communities, based on Shannon diversity indices

Source of variance	df	16 s rRNA		ITS	
		MS	F	MS	F
SP	4	6.06	24.57**	4.36	33.03**
CF	1	10.55	42.77**	0.53	3.98*
SP× CF	4	0.40	1.62	0.29	2.19
Residue		0.25		0.13	

‘SP’ denotes sampling compartments, and ‘CF’ represents continuous cropping. The terms ‘df’, ‘MS’, and ‘F’ stand for degrees of freedom, mean square, and F-value, respectively. The symbols ‘^*^’ and ‘^**^’ indicate significance levels of *P* < 0.05 and *P* < 0.01, respectively.

**Figure 2:**
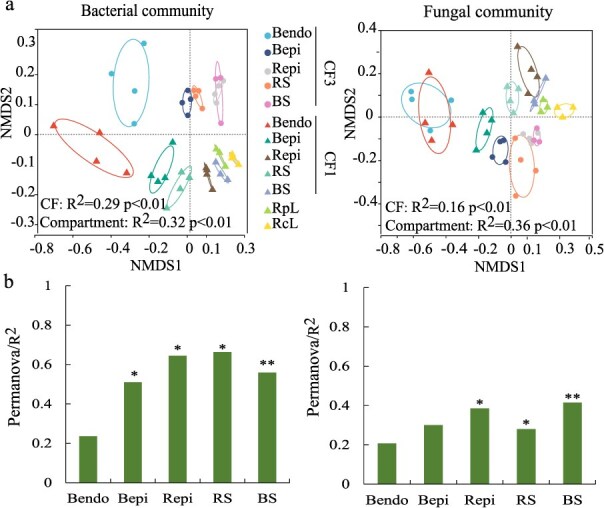
Composition of bacterial and fungal communities in Edible Lily. (a) Non-metric Multi-Dimensional Scaling (NMDS) ordinations of Bray–Curtis dissimilarity matrices with permutational analysis of variance (PERMANOVA), demonstrating significant associations between community composition of bacteria (left) and fungi (right) with continuous cropping (CF) and sampling compartment CF1 and CF3, sampled after 1 and 3 years of continuous edible lily cropping. RcL and RpL, bulk soils planted with rice and rapeseed after 3 years of continuous edible lily cropping, respectively. (b) Contributions of continuous cropping (CF) to the variation in bacterial (left) and fungal (right) community compositions in individual compartments, based on PERMANOVA. Significant levels are indicated by asterisks (^*^: *P* < 0.05, ^**^: *P* < 0.01).

**Figure 3:**
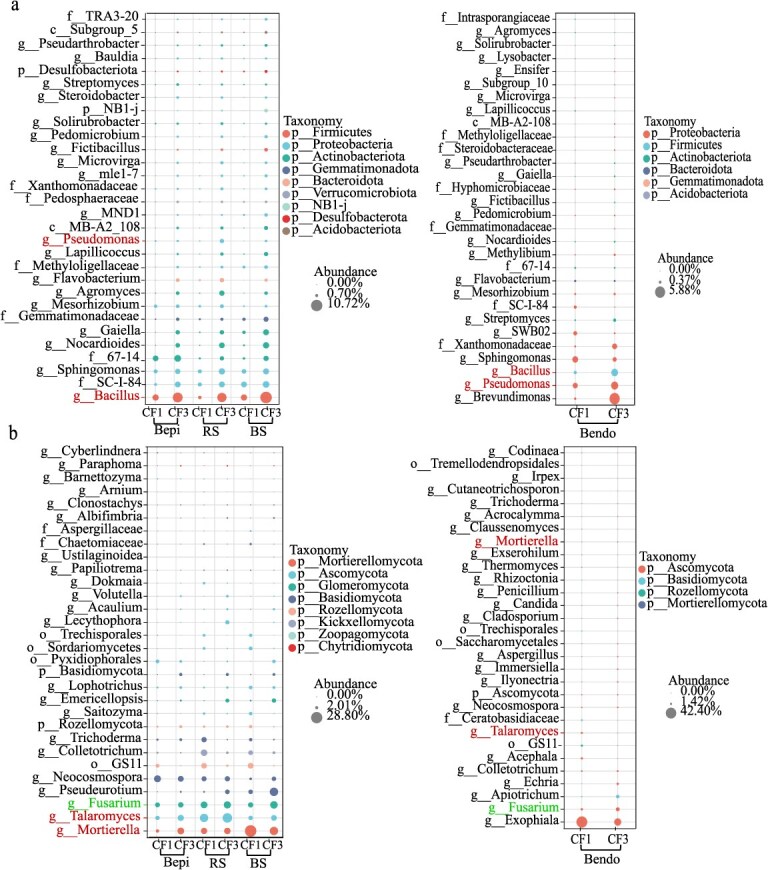
ANOVA analysis of the differences in the relative abundance of bacterial (a) and fungal (b) communities at the genus level in soils planted with edible lily for 1 year (CF1) and 3 years (CF3). Panel a details the comparisons for bacterial communities, while panel b focuses on the fungal communities. The analysis considered various soil compartments: bulk soil (BS), rhizosphere soil (RS), root episphere (Repi), bulb episphere (Bepi), and the endosphere (Bendo).

NMDS-PERMANOVA identified sampling compartment as the primary driver of microbiome variation (bacteria: *R*^2^ = 0.32; fungi: *R*^2^ = 0.36, *P* < 0.01), followed by CF (bacteria: *R*^2^ = 0.29; fungi: *R*^2^ = 0.16, *P* < 0.01) (Fig. 2a). Notably, CF exerted stronger effects on rhizosphere communities than bulb endosphere (Fig. 2b). Furthermore, both bacterial and fungal community structures in the RcL and RpL treatments closely resembled those in CF1, while exhibiting clear separation from CF3 (Fig. 2a). Differential abundance analysis highlighted CF-induced enrichment of beneficial bacteria (e.g. *Pseudomonas*, *Bacillus*) and dual-functional fungi (beneficial *Talaromyces*/*Mortierella* vs pathogenic *Fusarium*) in both rhizosphere and endosphere (*P* < 0.05, Fig. 3).

### Co-occurrence network dynamics under continuous cropping

**Figure 4:**
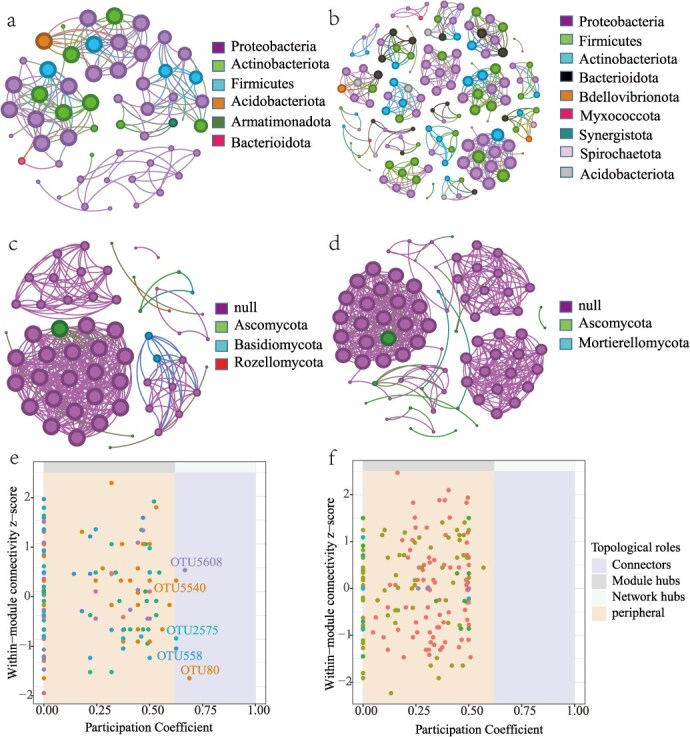
Co-occurrence network analysis based on Spearman's correlation analysis for connectivity and module partitioning based on selected OTUs. The network of endogenously bacterial community in CF1 (a) and CF3 (b), and fungal community in CF1 (c) and CF3 (d). The Zi-Pi plot of endogenously bacterial (e) and fungal (f) community based on their topological roles.

Endophytic networks in CF3 exhibited greater complexity than CF1, with increased edge numbers and average clustering coefficients (Fig. 4, Table S2). While bacterial networks maintained dominant *Proteobacteria*/*Actinobacteriota* hubs, including keystone taxa represented by OTUs such as Burkholderia-OTU2575 and Pseudomonas-OTU558, fungal networks lacked comparable hub taxa (Fig. 4e and f). Intriguingly, CF3 bacterial networks showed reduced positive correlation ratios (69% vs CF1's 99%), suggesting cropping duration modulates microbial interaction types.

### Rhizosphere-to-endosphere microbial recruitment

**Figure 5:**
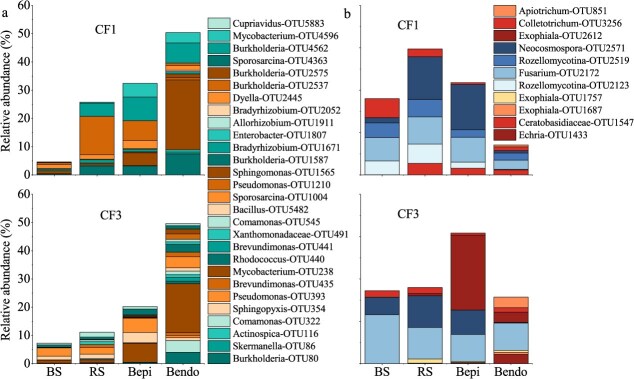
The recruitment of endophytes of lily bulb from rhizosphere soil microbes. The relative abundance of these co-occurrence bacteria (a) and fungi (b) under soil planting edible lily 1 year (CF1) and 3 year (CF3). BS, the bulk soil. RS, rhizosphere soil. Repi, root episphere. Bepi, bulb episphere. Bendo, endosphere. OTUs are denoted as Genus-OTUXXXX (e.g. Sporosarcina-OTU1004).

Compartmental filtering was evident along the BS → RS →Bepi→Bendo continuum (Fig. 2a). Bacterial OTU retention rates increased from rhizosphere (CF1:60%; CF3:69%) to bulb episphere (CF1, 73%; CF3, 79%), but sharply declined in endosphere (CF1, 23%; CF3, 35%) (Fig. S2a). Notably, CF3 enhanced recruitment of beneficial taxa (20 OTUs vs CF1's 12), with *Mycobacterium*-OTU238 dominating endosphere communities (~20% abundance, Fig. 5a). Fungal recruitment proved more selective, with only 10% of bulb endosphere reads originating from soil. Pathogenic *Fusarium*-OTU2172 showed CF3-associated enrichment (6.44% vs CF1's 2.18%), highlighting potential disease risk under prolonged cropping (Fig. 5b).

### Endophyte-mediated pathogen suppression

**Figure 6:**
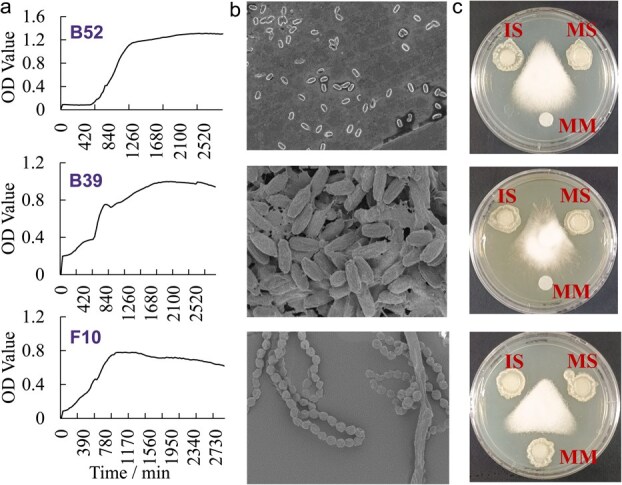
Morphological characteristics of core strains with antagonistic effects. Growth curve (a) and scanning electron microscope image (b) of core strains. (c) Test of antibacterial substances, analysing the effect of intracellular substances (IS), microbial solution (MS), and microbial metabolites (MM) against *Fusarium oxysporum*.

From the 69 initial isolates screened, 28 endogenous strains (19 bacterial and 9 fungal, including *Fusarium oxysporum*; Fig. S3a and b) were identified via 16S/18S rRNA sequencing. Antagonism assays using plate confrontation classified these strains as follows: three core strains (>50% mycelial inhibition: *Methylobacterium brachiatum* B39, *R. rhizogenes* B52, *Talaromyces* sp*.* F10); seven auxiliary strains (20–50% inhibition: bacterial strains *Rhizobium* sp*.* B30, *Mycobacterium* sp*.* B32, *Caulobacter* sp*.* B46, *Shinella* sp*.* B53; fungal strains *Penicillium glabrum* F1, *Pyronema omphalodes* F5, *Ogataea methanolica* F8); and seventeen strains discarded due to low inhibition (<20%; Fig. S3c and d). SynComs were constructed using strains meeting three criteria: (i) consistent antagonism (core strains), (ii) growth kinetic complementarity (bacterial μmax = 15.5–20.5 h vs fungal μmax = 27.5 h, Fig. 6), and (iii) synergistic compatibility (mutually antagonistic strains B53/B30/F8 excluded based on cross-streak assays showing growth inhibition; Fig. S4). Fungal metabolites showed superior inhibition (Fig. 6), and crucially, fungal-integrated SynComs (SCII/IV/V) outperformed bacterial-only consortia (SCI/III), achieving 65% *in vitro* inhibition and 25% plant biomass increase (Figs S5 and S6). Pot trials also demonstrated a significant reduction in disease severity (*P* < 0.05), with SynCom V (SCV) reducing the mean disease index to 43.82% ± 9.80% compared to 67.63% ± 9.12% in the SynCom III (SCIII) control group, representing a 35.2% reduction (Fig. S6).

## Discussion

### Continuous cropping reshapes microbial dynamics in lily ecosystems

Our findings demonstrate that continuous cropping enriches *Fusarium* pathogens in both lily endospheres and rhizospheres, corroborating previous reports of pathogen accumulation in monoculture systems [23]. Notably, however, we reveal a significant co-enrichment phenomenon: alongside pathogens, host plants recruit potentially beneficial taxa (e.g. *Pseudomonas*, *Bacillus*, *Talaromyces*, *Mortierella*) under continuous cropping pressure. This aligns with the ‘cry for help’ strategy observed in other systems like peppers [24].

However, a critical divergence from prior models emerges: The recruitment of these putative antagonists does not translate to effective suppression of *Fusarium* populations in this lily system. The observed co-enrichment suggests several non-exclusive possibilities: (i) The abundance or antagonistic activity of the recruited beneficial microbes may be insufficient to overcome pathogen pressure; (ii) their enrichment may primarily reflect a general plant stress response rather than specific, effective pathogen antagonism [13]; or (iii) some recruited antagonists may enter a functionally latent state or become decoupled from disease outcomes under the specific selective pressures of continuous cropping [25]. Therefore, the coexistence of enriched pathogens and recruited beneficial taxa likely represents a complex, context-dependent state rather than a simple ‘dynamic equilibrium’ implying effective counterbalance. This challenges assumptions that antagonistic microbiota invariably reduce pathogen loads [26] and highlights the need to assess not just microbial composition but also functional activity and ecological interactions in situ.

Furthermore, continuous cropping significantly reshaped microbial interactions, increasing network complexity in both bacterial and fungal communities while reducing the proportion of positive bacterial correlations. This supports the hypothesis that environmental stress drives functional diversification and niche partitioning [27], but contrasts with studies emphasizing mutualistic networks stability as key to disease suppression [28]. The prevalence of negative correlations may reflect intensified competitive exclusion acting to stabilize communities under stress—a mechanism previously theorized [29] and now empirically demonstrated in perennial crops system. This shift towards competition could further contribute to the observed lack of pathogen suppression despite antagonist enrichment.

Interestingly, the pronounced structural convergence of bacterial and fungal communities in RcL/RpL with CF1, alongside distinct divergence from CF3 (Fig. 2a), indicates that rotations initiate a partial microbiome rehabilitation. This likely stems from the host-specific recruitment of beneficial taxa mediated by rice or rapeseed root exudates [30], which mitigates—though does not fully reverse—community degeneration induced by extended monoculture. Crucially, this observed structural shift towards CF1-level composition suggests rotational crops disrupt pathogen enrichment cycles characteristic of continuous lily cropping [31], offering practical phytosanitary benefits despite the unaltered alpha diversity metrics.

### Host-driven filtering shapes a protective yet dynamically balanced endophytic niche

A key innovation of this study lies in quantifying hierarchical microbiome assembly. While 50% of endophytic bacteria originated from soil, their enrichment from bulk soil → rhizosphere → bulb interior underscores stringent host filtering [32]. Surprisingly, despite *Acidobacteria*'s known plant-beneficial roles [33], these taxa were depleted in lily endospheres. This selective exclusion—contrasting with rice and wheat studies [34]—implies crop-specific filtering priorities, potentially favoring antimicrobial producers like *Mycobacterium*. The dominance of soil-derived *Mycobacterium* in endospheres (a novel observation in Lilaceae) highlights its ecological versatility, possibly through antimicrobial synthesis or niche competition [35]. Critically, this host-driven filtering under continuous cropping pressure resulted in the co-enrichment of both putatively antagonistic microbes (*Pseudomonas*, *Bacillus*, *Talaromyces*, *Mortierella*) and the pathogen *Fusarium*. This suggests the selected community exists in a dynamic equilibrium where recruited antagonists are insufficient—either in abundance, functional activity, or timely deployment—to fully suppress pathogen proliferation, or where their antagonistic potential may become decoupled from disease outcomes under chronic stress. Host filtering disproportionately affected bacteria over fungi, likely due to differing dispersal capacities and metabolic integration [36]. This finding challenges the uniform application of microbiome assembly theories across microbial kingdoms and emphasizes the need for taxon-specific models in crop management. The observed co-enrichment underscores that while host selection favors potentially protective microbes, the natural equilibrium achieved under continuous cropping is sub-optimal for disease control, necessitating targeted interventions like SynComs.

### Synthetic communities: from concept to field-translational challenges

Building on the isolated antagonists (*Rhizobium*, *Methylobacterium*, *Talaromyces*), we innovatively developed SynComs that outperformed single-strain treatments—a critical advance beyond conventional biocontrol approaches [37]. Fungal-containing SynComs showed particular efficacy, likely due to niche overlap with *Fusarium* enhancing resource competition [3]. This aligns with but extends Gao *et al*. [24], demonstrating that fungal SynCom stability, not just sensitivity, drives pathogen suppression.

Our co-occurrence network analysis revealed that continuous cropping significantly altered the in-situ network topology (Fig. 4). While we did not directly identify specific keystone taxa (e.g. connectors or module hubs) within the designed SynComs, it raises a fundamental question: does the successful SynCom, particularly bacterial–fungal integration SC V, recapitulate key architectural features of a healthy, suppressive soil network? Although not empirically confirmed here, we propose that its superior performance may, in part, be explained through a network theory lens. The inclusion of multi-kingdom members potentially builds ecological complexity and redundant interactions that enhance community resilience and pathogen suppression, mirroring principles of functional soil microbiomes [38, 39]. This theoretical framework offers a mechanistic hypothesis for why a diverse consortium could be more effective than single strains.

While laboratory efficacy is clear, field applicability remains unproven—a limitation shared by most SynCom studies [40]. Environmental variability and resident microbiome resistance may hinder translational success, necessitating formulation optimizations (e.g. carrier materials, application timing). Nevertheless, our work provides the first evidence for SynCom-based *Fusarium* control in lilies, bridging the gap between microbiome theory and sustainable horticulture. Crucially, we identify host filtering as a tunable lever for microbiome engineering—a paradigm shift from environment-focused amendments. The efficacy of a SynCom depends not just on its inherent composition but also on its ability to successfully colonize and engage with the host. We hypothesize that the superior performance of SC V could be partly due to the fungal members priming the plant's immune system (Induced Systemic Resistance, ISR), thereby fostering a more active host defense [41], although this specific mechanism requires further validation.

## Conclusions

This study demonstrates the ecological promise of synthetic microbial communities (SynComs) for sustainable soil-borne disease control in lily cultivation. Through metacommunity analysis of rhizosphere and bulb microbiomes under continuous cropping, we identified enriched core taxa including beneficial bacteria (*Pseudomonas*, *Bacillus*) and fungi (*Talaromyces*, *Mortierella*) co-enriched with the pathogen *Fusarium oxysporum*. While this enrichment signifies a host response to pathogen stress, it reflects a dynamic equilibrium where these antagonists are naturally insufficient or potentially decoupled from effective biocontrol outcomes under continuous cropping pressure. Fungal-integrated SynComs outperformed bacteria-only consortia in suppressing *Fusarium oxysporum* and promoting plant growth, attributable to niche competition and enzymatic synergy. While specific keystone taxa were not directly identified, this enhanced performance may reflect a network-level resilience resembling that of a healthy, suppressive soil microbiome. These results advance host-mediated microbiome engineering, positioning SynComs as dual-function tools for soil health restoration and agrochemical reduction. While promising, field application requires optimization to address environmental variability and microbial competition. Future work should focus on field validation of SynCom persistence, mechanistic dissection of cross-kingdom interactions, and host-specific endophyte recruitment. This research bridges microbiome science and sustainable agriculture, offering a framework for productivity-ecosystem resilience balance.

## Materials and methods

### Experimental design and site description

The field experiment was conducted in Longshan County (29°36′6′′N, 109°35′10′′E; elevation 541 m), Hunan Province, China, characterized by shale-derived soils and a subtropical humid monsoon climate (mean annual temperature: 15.8°C; precipitation: 1400 mm; frost-free period: 270–280 days). Four treatments were established in a randomized complete block design with four biological replicates per treatment (*n* = 4): RcL, rice rotation after 3-year continuous lily cultivation; RpL, rapeseed rotation after 3-year continuous lily cultivation; CF1, first-year lily monoculture; CF3, third-year continuous lily monoculture. Each treatment contained four replicate plots (3 m × 6 m), with the local cultivar *Lilium lancifolium cv.* Juandan cultivated following regional agronomic practices.

### Sample collection and processing

Sampling was conducted during October 2021 at physiological maturity. Five microbial compartments were investigated (Fig. 1a):

Bulk soil (BS): Collected from 0–20 cm depth at points located at least 20 cm away from plant roots;

Rhizosphere soil (RS): Soil adhering to roots recovered by manual shaking [2, 42];

Root episphere (Repi): Surface microbiota isolated via ultrasonic dispersion (40 Hz, 90 W, 5 min);

Bulb episphere (Bepi): Surface microbiota from sterilized bulbs;

Bulb endosphere (Bendo): Internal tissue microbiota isolated from surface-sterilized bulbs (sequential sterilization: 75% ethanol, 1.2% NaClO, followed by sterile distilled water rinse).

Five plants per plot were sampled, with all samples were immediately frozen in liquid nitrogen and stored at −80°C [43, 44].

### Analysis of soil properties

Collected soil samples were stored at 4°C prior to analysis ammonium-N (NH_4_^+^-N), nitrate-N (NO₃^−^-N), and dissolved organic carbon (DOC) according to Lu [45] and Qin *et al*. [46]: NH_4_^+^-N and NO₃^−^N were extracted with 1 M KCl and analysed using a FIAstar 5000 continuous flow injection analyser (Foss); DOC was extracted with 0.5 M K_2_SO_4_ and quantified as total oxidizable carbon using a TOC-VMP analyser (Shimadzu). For the analysis of available phosphorus (AP), available potassium (AK), total carbon (TC), total nitrogen (TN), total phosphorus (TP), total potassium (TK), and pH (following Lu [45]), soils were air-dried and sieved (<2 mm). AP was extracted with ammonium fluoride-hydrochloric acid and measured by molybdenum-antimony colorimetry; AK was determined by flame photometry; TC was measured via the K_2_Cr_2_O_7_ oxidation method; TN was analysed using an AA3 continuous flow analyser after H_2_SO_4_ digestion; TP was determined by the molybdophosphate method following digestion with HClO_4_–H_2_SO_4_; TK was measured by flame photometry after NaOH fusion; soil pH was determined potentiometrically at a 1:2 soil/water ratio.

### DNA extraction and high-throughput sequencing

Total DNA was extracted using: Soils, fastDNA SPIN Kit (MP Biomedicals, USA); Plant tissues, E.Z.N.A. HP Plant DNA Kit (Omega, USA). Amplicon libraries targeting bacterial 16S rRNA (V5-V7 regions, primers 799F/1193R) and fungal ITS1 (primers ITS1F/ITS2R) were constructed [47, 48]. Sequencing was performed on an Illumina MiSeq platform (250 bp paired-end reads; Majorbio, China). Raw FASTQ files were demultiplexed and quality filtered using QIIME v1.8.0. All samples were normalized to a similar sequencing depth using MOTHUR. OTUs were clustered at 97% similarity using UPARSE v7.1 [49], and chimeric sequences were removed with UCHIIME. Representative sequences from each OTU were annotated for species identification using NCBI server and a search against type strains at a 70% confidence threshold [50]. OTUs are denoted as Genus-OTUXXXX (e.g. Burkholderia-OTU2575), where Genus is the assigned taxonomic genus and ‘OTUXXXX’ is a unique identifier. The number (e.g. 2575) serves only as a non-taxonomic ID.

### Synthetic microbial community construction

Endophytes were isolated from surface-sterilized bulbs through tissue homogenization and cultured on selective media. Bacteria: LB agar with 16S rRNA identification (27F/1492R primers); Fungi: PDA with 18S rRNA identification (NS1/NS4 primers). Antagonistic strains against *Fusarium oxysporum* were classified as: Core strains: >50% mycelial growth inhibition; Auxiliary strains: 20–50% inhibition. After screening for antagonistic interactions via pairwise plate confrontation assays, the selected strains were used to assemble a synthetic community (SynCom). Total five SynCom formulations (I-V) were prepared by combining equal volumes (10^8^ CFU/ml) of selected strains (Table S3) to systematically test the effects of microbial composition and complexity: Bacteria-only cores (SC I), fungi-only core (SC II), bacterial core+auxiliaries (SC III), fungal core+auxiliaries (SC IV), and full bacterial-fungal integration (SC V). Electron microscope images were taken using a field emission scanning electron microscope (Hitachi SU8010, Japan).

### Evaluation of biocontrol efficacy

The antagonistic activity of the endophytic SynCom against *Fusarium oxysporum* was assessed using a dual-culture plate confrontation assay [51] complemented by pot experiments. Antibacterial activity was determined via a modified filter paper disc diffusion method [25], wherein sterile filter paper discs (6 mm diameter) were positioned on PDA plates and impregnated with 50 μL aliquots of either intracellular extracts, microbial suspensions, or metabolites. All treatments were conducted in triplicate with appropriate sterile controls, followed by incubation at 30°C with orbital agitation (180 rpm) for 48 h. Metabolite extraction involved centrifugation of microbial suspensions (10 000 × *g*, 10 min), sterile filtration (0.22 μm) of supernatants, and ultrasonic disruption (250 W, 25 min) of pelleted cells prior to final filtration.

Soil for pot trials was collected from a commercial Lilium cultivation site in Longshan County with a documented three-year monoculture history. Following air-drying and sieving (5 mm mesh), the substrate was adjusted to 30% field capacity and dispensed into PVC containers (24 cm diameter × 25.5 cm height; 4.5 kg per pot). The experimental design comprised five SynCom formulations (detailed in Table S1) and a sterile water control (CK), arranged in a completely randomized design with four replicates per treatment (24 pots total). Three lily bulbs were planted per pot in October 2022, with SynCom inoculations (10 ml, ≥10^8^ CFU ml^−1^) administered during both seedling establishment and rapid growth phases. After six months, treatment efficacy was quantified through biometric measurements (plant height, leaf area) and disease indices (DI, leaf wilt index on 0–4 scale; vascular browning index according to [52]). The Disease Index (DI) for each treatment replicate was calculated as: DI (%) = [Σ (N_i_ × S_i_) / (N × S_max_)] × 100, where N_i_ = Number of plants/organs in severity grade i; S_i_ = Numerical value of grade i (0: healthy, 1: 1–25% symptoms, 2: 26–50%, 3: 51–75%, 4: 76–100%); N = total number of plants/organs assessed; S_max_ = Highest severity grade (e.g. 4). The mean DI ± standard error (SE) per treatment group was then calculated.

### Statistical analysis

All data were tested for normality prior to analysis. Univariate statistical analyses were performed using SPSS (v20.0, IBM Corp.) with one-way ANOVA and LSD post hoc tests (α = 0.05) to evaluate alpha diversity changes and SynCom biocontrol efficacy against *Fusarium oxysporum*. Multivariate analyses in R (v4.2.0) included: (i) beta-diversity assessment via NMDS ordination and PERMANOVA (Bray-Curtis distances, 999 permutations); (ii) redundancy analysis (RDA) of microbe-environment relationships with Monte Carlo testing (999 permutations); and (iii) differential abundance analysis using metagenomeSeq. Microbial networks were constructed from OTUs >0.1% abundance (R v3.6.3) using Spearman correlations (|ρ| ≥ 0.7, *p* < 0.01), with topological analysis in Gephi (v0.9.2) categorizing nodes into network hubs (Zi ≥ 2.5, Pi≥0.6), module hubs (Zi ≥ 2.5, Pi<0.6), connectors (Zi < 2.5, Pi≥0.6), and peripherals (Zi < 2.5, Pi<0.6) following Berry & Widder [53]. Phylogenetic reconstruction employed MEGA6 (maximum likelihood, Kimura-2 parameter model, 1000 bootstraps).

## Supplementary Material

Web_Material_uhaf286

## Data Availability

The raw high-throughput sequence data of the bacterial *16S rRNA* and fungal ITS genes have been deposited in the GenBank Sequence Read Archive under accession numbers PRJNA1155822 and PRJNA1155828, respectively. The raw sanger dideoxy sequence data of the bacterial *16S rRNA* and fungal *18S rRNA* genes reported in this paper were deposited in the GenBank under accession numbers PQ278707-PQ278760 and PQ276912-PQ276926, respectively.
