# Supplementary material for: Decoding endophytic microbiome dynamics: engineering antagonistic synthetic consortia for targeted fusarium suppression in monoculture regimes
Source: Hortic Res. 2025 Oct 16;13(2):uhaf286. doi: 10.1093/hr/uhaf286 (PMC12893865; doi:10.1093/hr/uhaf286)

**Table S1**

Chemical characterization of bulk soil samples. CF1 and CF3, sampled after 1 and 3 years of continuous edible lily cropping. RcL and RpL, bulk soils planted with rice and rapeseed after 3 years of continuous edible lily cropping, respectively. DOC, dissolved organic carbon; AN, alkali-hydrolyzable nitrogen; AP, available phosphorus; AK, available potassium; TN, total nitrogen; TP, total phosphorus; TK, total potassium. Numbers after ± are standard deviations, n = 4; Different letters within rows indicate significant difference (*p*< 0.05).

| Items | CF1 | CF3 | RcL | RpL |
| --- | --- | --- | --- | --- |
| DOC（mg/kg） | 256.46+4.02a | 144.09+14.07d | 213.01+8.71b | 232.88+8.49c |
| Nitrate-N（mg/kg） | 59.27+21.79a | 4.24+1.61b | 6.61+0.34b | 5.13+0.42b |
| Ammonium-N（mg/kg） | 28.96+8.62a | 2.53+1.31b | 1.26+0.20b | 2.97+2.30b |
| TP(g/kg） | 0.55+0.05b | 0.60+0.11ab | 0.64+0.05ab | 0.70+0.04a |
| AP（mg/kg） | 114.53+3.30a | 123.94+13.45a | 58.79+7.57b | 69.49+4.21b |
| AK（mg/kg） | 374.57+158.24a | 425.60+51.33a | 173.23+16.06b | 178.80+73.41b |
| TK（%） | 1.05+0.16a | 1.22+0.24a | 1.03+0.05a | 1.13+0.12a |
| TC（%） | 1.08+0.07a | 1.35+0.44a | 1.62+0.50a | 1.11+0.19a |
| TN（%） | 0.11+0.01a | 0.12+0.01a | 0.15+0.05a | 0.12+0.00a |
| pH | 4.91+0.74a | 4.40+0.22a | 4.78+0.06a | 4.44+0.03a |

**Table S2**

The topological characteristics of endogenously bacterial and fungal community network bacterial and fungal community network in lily bulb. ACC, Average Cluster Coefficient. Positive and Negative, the radio of edges with positive and negative correlations.

| Items | | Nods | Edges | Graph density | ACC | Positive | Negative |
| --- | --- | --- | --- | --- | --- | --- | --- |
| Bacteial endophtes | CF1 | 54 | 152 | 0.11 | 5.63 | 98.68 | 1.32 |
|  | CF3 | 139 | 406 | 0.04 | 5.84 | 68.72 | 31.28 |
| Fungal endophtes | CF1 | 58 | 333 | 0.20 | 11.5 | 90.39 | 9.61 |
|  | CF3 | 81 | 466 | 0.14 | 11.5 | 97.85 | 2.15 |

**Table S3**

Components of synthetic microbial community formulations

| SynCom formulations | | Bacterial core strains | Fungal core strains | Auxiliary strains |
| --- | --- | --- | --- | --- |
| SCⅠ | Bacterial core strains | B52、 B39 | — | — |
| SCⅡ | Fungal core strains | — | F10 | — |
| SCⅢ | Bacterial core + auxiliaries strains | B52、 B39 | — | B46、B32、F1、F5 |
| SCⅣ | Fungal core + auxiliary strains | — | F10 | B46、B32、F1、F5 |
| SCⅤ | Composite core + auxiliary strains | B52、 B39 | F10 | B46、B32、F1、F5 |

**Fig S1**

Redundancy analysis (RDA) was conducted to explore the relationship between bacterial (a) and fungal (b) communities and soil chemical properties. The properties analyzed included total nitrogen (TN), total phosphorus (TP), total potassium (TK), dissolved organic carbon (DOC), available phosphorus (AP), and available potassium (AK). Bulk soil planting edible lily 1 year (CF1) and 3 year (CF3). RcL and RpL, bulk soils planted with rice and rapeseed after 3 years of continuous edible lily cropping, respectively.


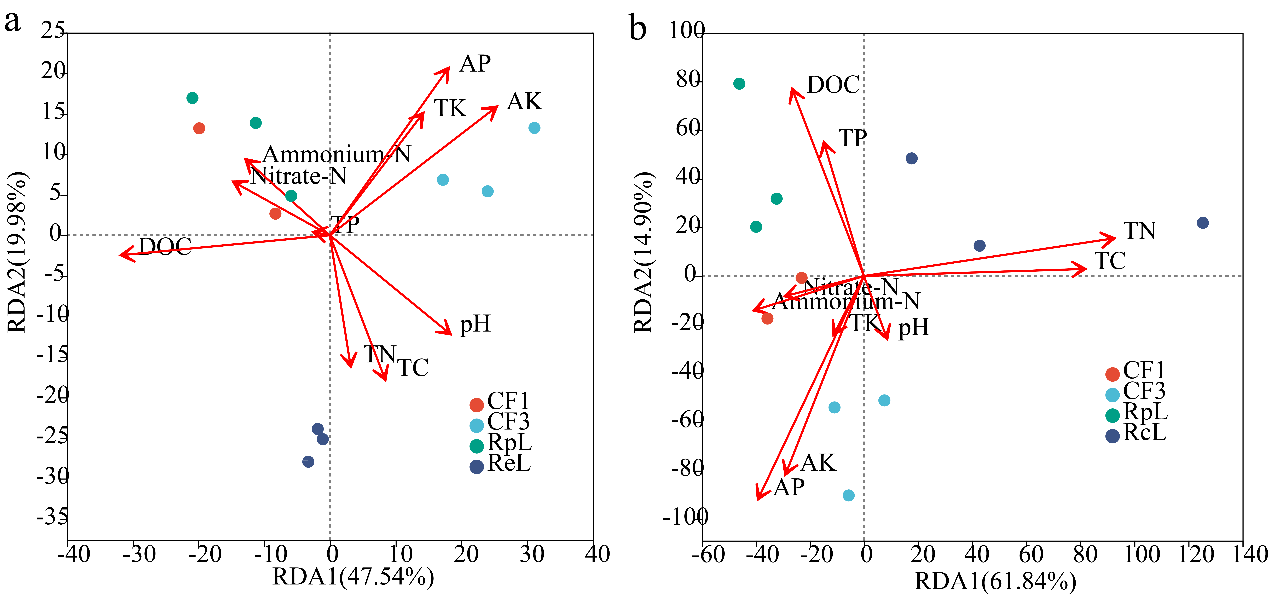


**Fig. S2**

The recruitment of endophytes of lily bulb from rhizosphere soil microbes. The bacterial (a) and fungal (b) number of OTUs in co-existed in BS, RS, Bepi and Bendo, step by step under soil planting edible lily 1 year (CF1) and 3 year (CF3). BS, the bulk soil. RS, rhizosphere soil. Repi, root episphere. Bepi, bulb episphere. Bendo, endosphere.


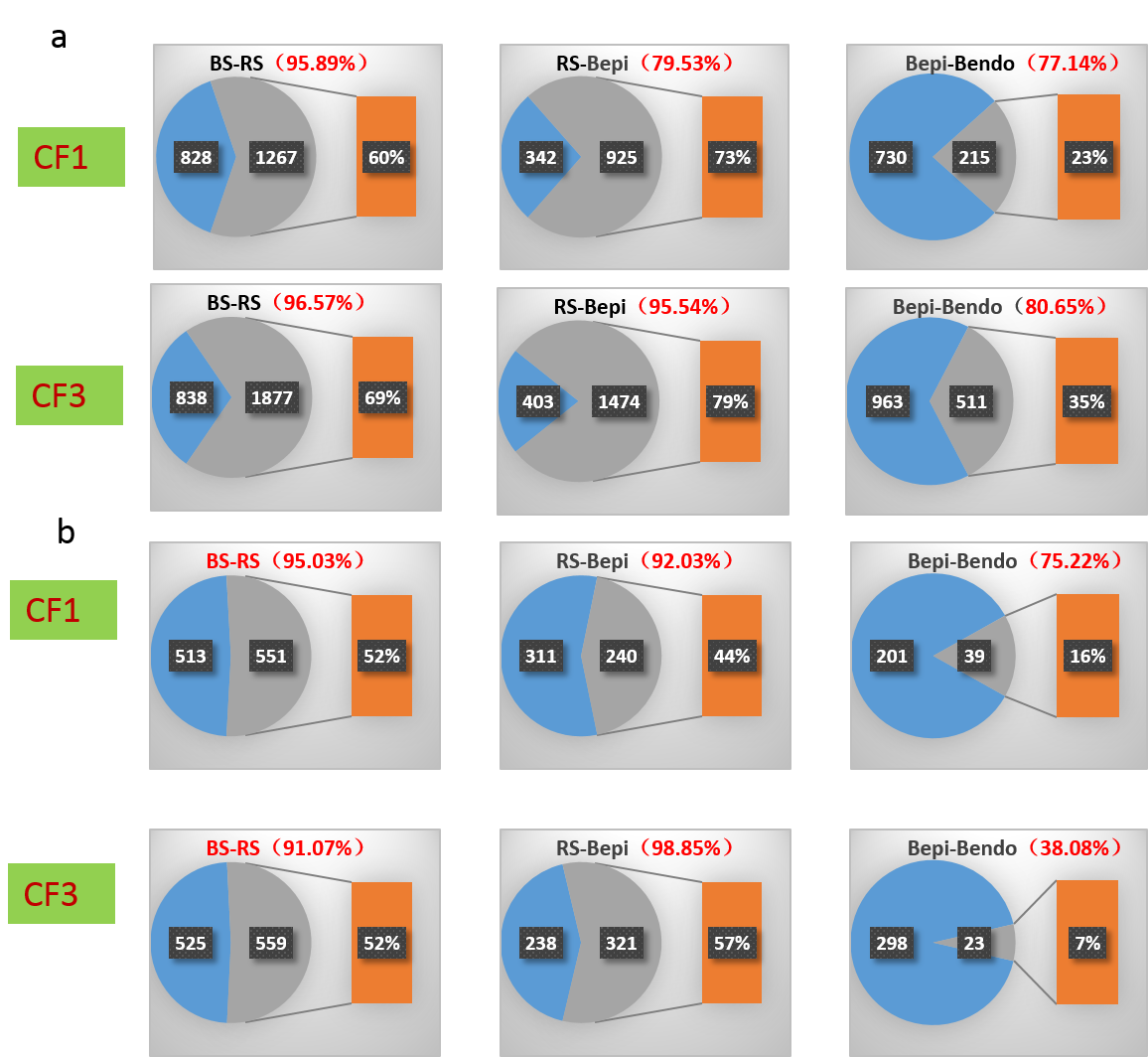


**Fig S3**

Antagonistic effects of endophyte isolates against Fusarium oxysporum. (a-b) Phylogenetic dendrograms of endophytic bacterial (a) and fungal (b) isolates. (c-d) Antibacterial effects of endophytic bacterial isolates (c) and fungal isolates (d) against the pathogen *Fusarium oxysporum*, with strains exhibiting significant antagonistic effects marked in red.

**
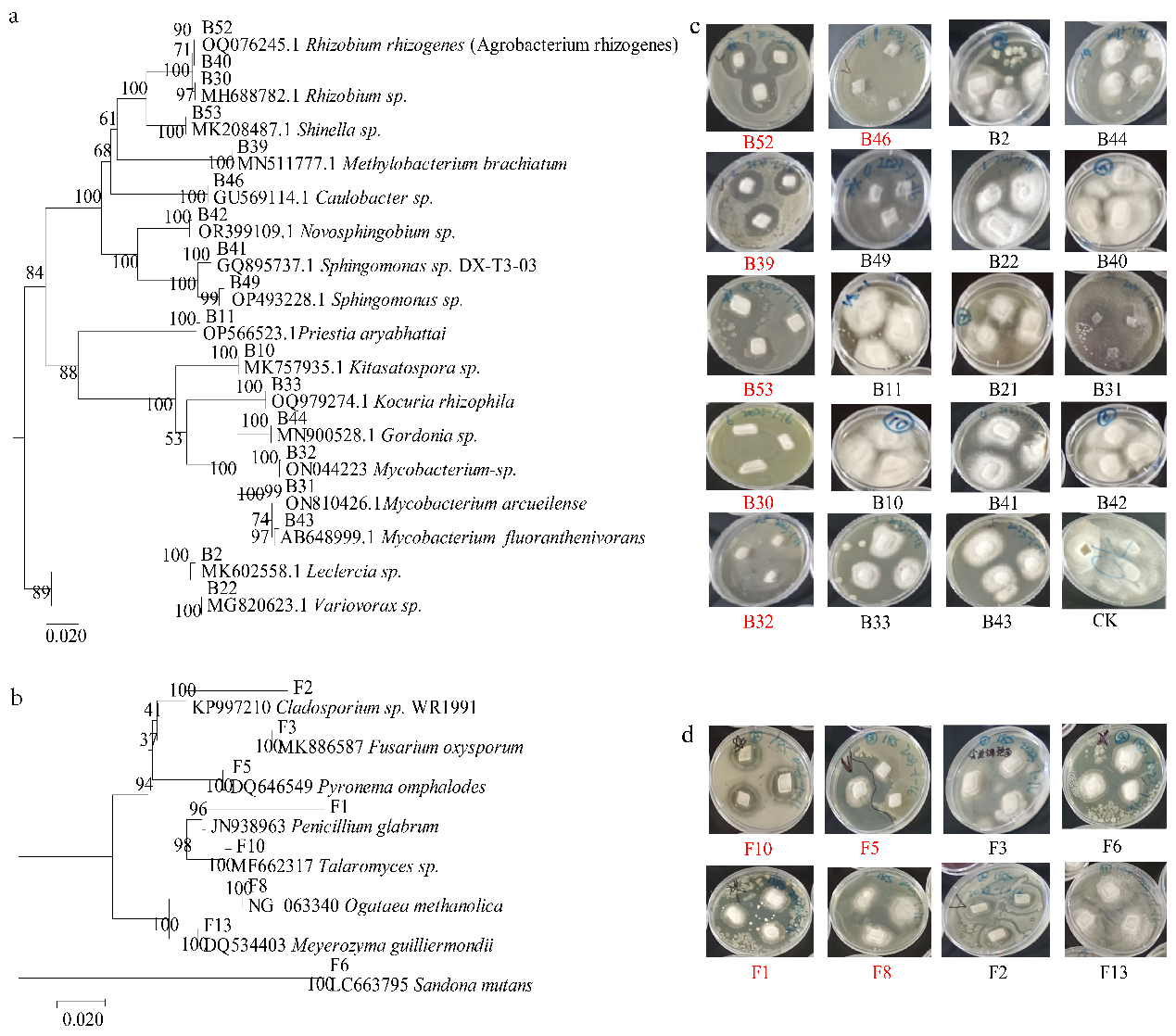
**

**Fig S4**

A paired flat panel confrontation test was conducted to evaluate the antagonistic effects between endophytic bacterial and fungal isolates. In the test results, strains exhibiting obvious antagonistic effects were marked in red. This method allows for the direct observation of interactions between different microbial strains, identifying those capable of inhibiting the growth of their counterparts.


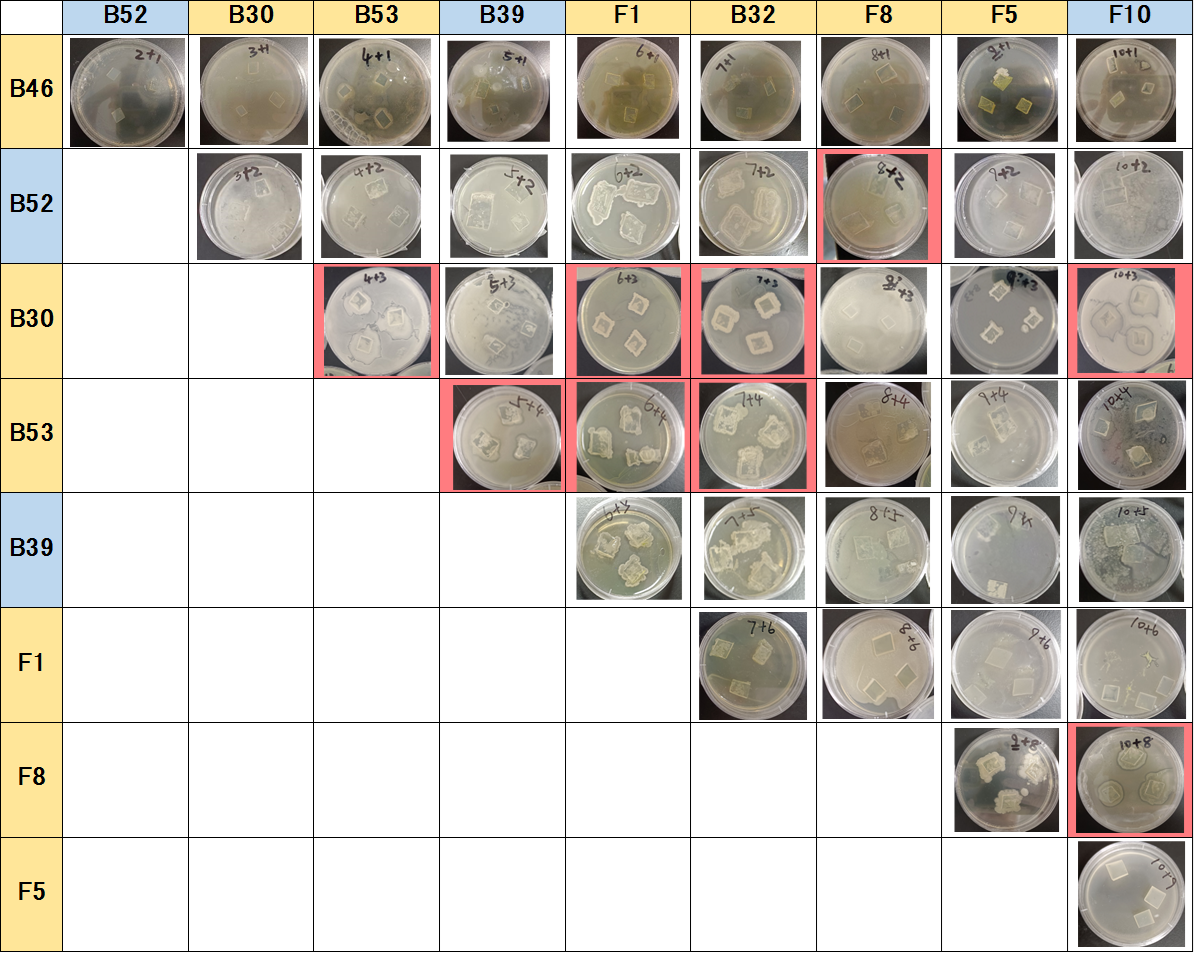


**Fig S5**

The antibacterial effects of single microbes and synthetic microbial communities (SynComs) against the pathogen *Fusarium oxysporum*.


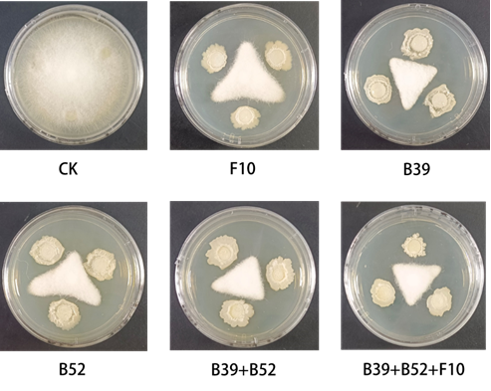


**Fig S6**

Impact of synthetic microbial communities (SynComs) on Plant Growth. (a) Phenotypes of edible lilies after six months of growth in a greenhouse, inoculated with SynComs. (b) Effects of different SynComs on plant height, plat width and disease indices, comparing controls with sterile setups (CK) and SynComs with various combinations of core strains of bacteria, fungus, and auxiliary strains (SCⅠ to SCⅤ). Data are shown as mean values with standard errors (N = 4), and lowercase letters indicate significant differences at *p* < 0.05 among the different SynComs.


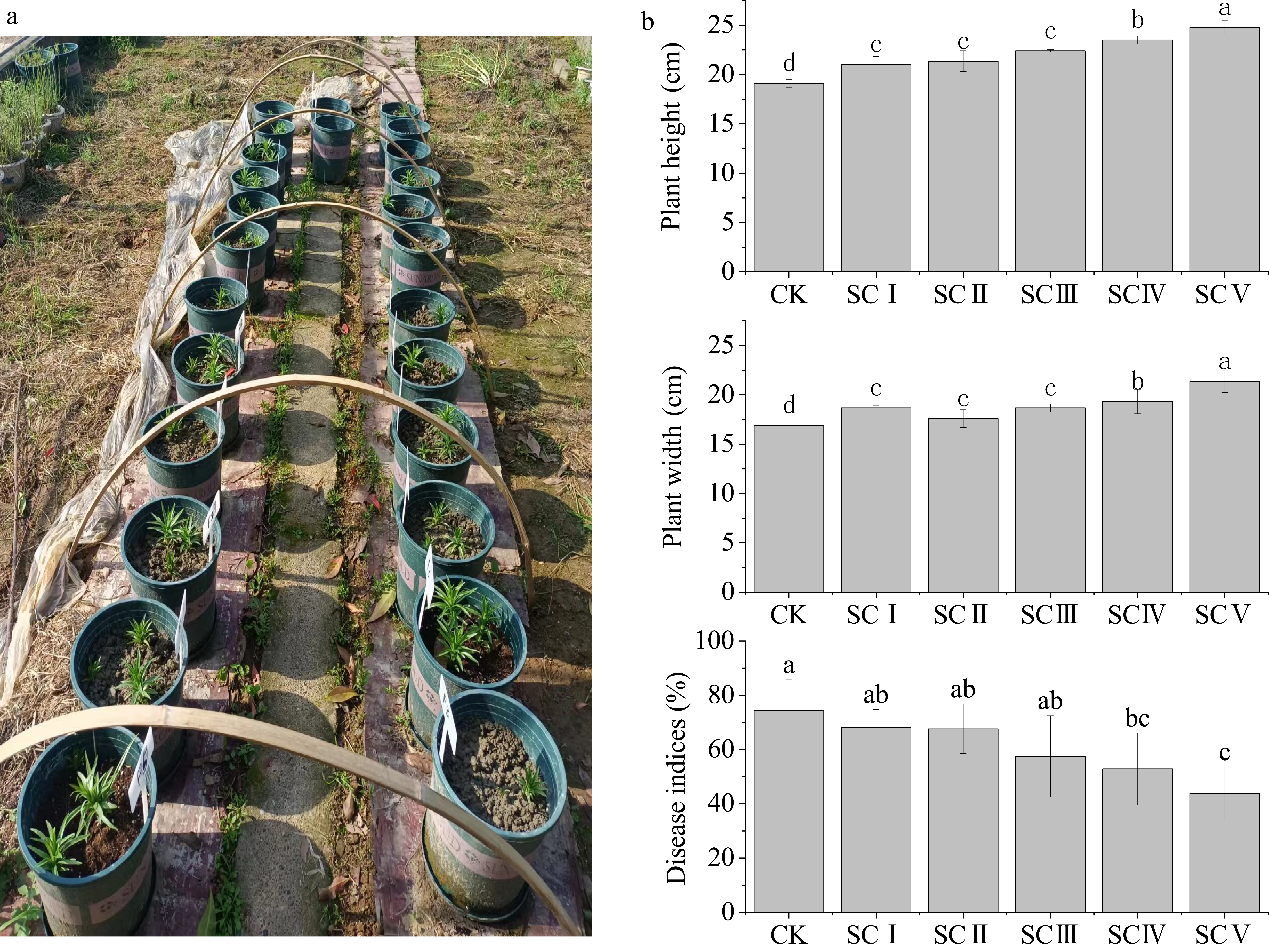

Supplement: Web_Material_uhaf286 [file web_material_uhaf286.zip › Clean version of revised suppl_imformation.docx]
